# Sociodemographic trends associated with caesarean delivery in rural subdistricts of Bangladesh: a cohort study, 2005–2019

**DOI:** 10.1136/bmjgh-2024-018600

**Published:** 2025-12-19

**Authors:** Anisur Rahman, Kimberly Clair, Monjur Rahman, U Tin Nu, Shaki Aktar, Bidhan Krishna Sarker, Fatema Khatun, Jesmin Pervin, Abdur Razzaque, Randall Kuhn

**Affiliations:** 1 Maternal and Child Health Division, International Centre for Diarrhoeal Disease Research, Bangladesh (icddr,b), Dhaka, Bangladesh; 2 Department of Women's and Children’s Health, Global Health and Migration Unit, Uppsala University, Uppsala, Sweden; 3 Center for the Study of Healthcare Innovation, Implementation, and Policy (CSHIIP), US Department of Veterans Affairs, Los Angeles, CA, USA; 4 Health Systems and Population Studies Division, International Centre for Diarrhoeal Disease Research, Bangladesh (icddr,b), Dhaka, Bangladesh; 5 Department of Community Health Sciences, Fielding School of Public Health, University of California, Los Angeles, Los Angeles, California, USA

**Keywords:** Maternal health, Global Health, Epidemiology

## Abstract

**Introduction:**

Although an appropriate population range for caesarean section (CS) has been debated, the WHO recommends national CS rates be between 10% and 15%. Yet rates are much higher in many low-income and middle-income countries, including Bangladesh. These studies also reveal wide disparities in CS use by geographic location and sociodemographic conditions. Nevertheless, few studies have the sample size and temporal precision necessary to model convergence and divergence trends over time. We assessed trends in CS segregated by key sociodemographic factors known to affect CS.

**Methods:**

We conducted our study using prospective pregnancy observation data from two distinct Maternal, Neonatal, and Child Health service areas: International Centre for Diarrhoeal Disease Research, Bangladesh (icddr,b) and the government service areas in Matlab, Bangladesh. We evaluated sociodemographic variations and trends using the multivariable logistic regression model.

**Findings:**

The proportion of CS rose from 5.9% in 2005 to 55.7% in 2019, about a ninefold increase, implying a 3.3% average annual growth rate. The CS was positively associated with residence in the icddr,b service area (vs government service area), older age, higher assets, lower parity births and, to a lesser extent, with higher levels of schooling. Among factors positively associated with CS, we observed negative time interactions for maternal age, education and place of delivery, suggesting a convergence in CS rates between high-risk and low-risk groups in these categories. However, positive time interactions for parity and asset score indicate that disparities in CS rates persist and are widening over time.

**Conclusions:**

Transition towards near-universal CS, irrespective of sociodemographic status or service area, is likely to be the inevitable future for rural Bangladesh. If such high rates of CS are considered sub-optimal for mother-and-child health, then evidence is needed to support alternatives.

**WHAT IS ALREADY KNOWN ON THIS TOPIC**

- Caesarean section (CS) rates are much higher than the WHO-recommended rate of 10–15% in many low-income and middle-income countries, including Bangladesh.
- Studies in different countries have shown strong associations between maternal sociodemographic factors and the proportion of CS.

**WHAT THIS STUDY ADDS**

- This study provides the clearest evidence of the rapid transition towards nearly universal CS delivery in a rural area of Bangladesh.
- Among factors positively associated with CS, we observed negative time interactions across age groups, education level and types of service areas; however, for the asset score, a positive interaction was observed.

**HOW THIS STUDY MIGHT AFFECT RESEARCH, PRACTICE OR POLICY**

- New studies are needed to establish the potential health risks associated with these high patterns of CS practices and to understand the potential social and institutional factors influencing this trend.

## Introduction

 Caesarean section (CS) is a surgical procedure to deliver babies through incisions on the uterus and abdominal wall.^1^ It has proven to be an effective obstetric procedure to prevent fetal, neonatal and maternal mortality in cases of a medical emergency.^2 3^ At a population level, CS procedures are often used to measure the quality and level of access women have to advanced obstetric care services.^2 4^ Although an appropriate population range for CS has been debated, the WHO recommends that country-wide CS rates be between 10% and 15% and that proportions outside this percentage are considered inadequate or medically unnecessary.^3^ A rate below 10% indicates a significant portion of women lack access to surgical obstetric care, and rates above 15% indicate overutilisation of this procedure for reasons other than medical necessity.^3^ The world has surpassed the WHO recommendation, with roughly 20% of births worldwide performed via CSs.^2 5^ Medically unnecessary CS has negative health consequences for mothers and their children.^6 7^ In 2008, a WHO survey of 373 facilities across 24 countries confirmed that unnecessary caesareans are associated with an increased risk of maternal morbidity and mortality compared with natural delivery.^8^ Additional research has established an association between caesareans and a heightened risk of placenta previa, placenta accreta and postpartum sepsis in future pregnancies.^6 7 9 10^ Furthermore, a systematic review that mostly includes studies from sub-Saharan Africa reports CS is associated with postpartum morbidity and readmission.^11^

In Bangladesh, the rates of scheduled caesareans have risen exponentially across all age groups over the past decade. Between 2004 and 2018, the national CS rate increased from 3.5% to 33%, about a 10-fold increase, according to the Bangladesh Demographic and Health Survey.^12 13^ In fact, data show that 70% of all caesareans performed in Bangladesh are elective or not medically necessary to save the mother’s or child’s life.^14^ Unnecessary CS can also lead to increased health expenditures at the family and societal levels. Recent data show that the economic burden of CS on families is substantial.^15 16^

Several studies examining the sociodemographic characteristics that likely determine CS use in Bangladesh produced similar results: women of high economic and educational status are more likely to deliver their babies via CS.^17^,^19^ Furthermore, CS rates are higher in urban health centres as compared with rural areas.^18 19^ Yet data on the individual social and demographic determinants of CS are sparse, often depending on unrepresentative clinical samples or retrospective reporting in Demographic and Health Surveys.^19^

The present study explores the rise of CS over a decade of a sharp increase in Matlab, a rural area of Bangladesh that has been the site of continuous demographic innovation through its Maternal, Newborn, and Child Health (MNCH) and Family Planning interventions. Matlab also has an ongoing prospective demographic registration of all vital events every 2 months, with the recording of CS delivery since 2005.^20 21^ The large and representative samples of births for a rural area over a long period allow us to visualise temporal trends in the drivers of CS. Specifically, we inquire to what extent variations in the prevalence of CS by wealth, education, age and parity are widening or narrowing over time. By separating trends for forerunner groups such as the wealthy from other groups, we may see signs of decelerating CS growth as rates reach a societal maximum.

## Methods

### Study setting

This cohort study was conducted in Matlab North and Matlab South, the two rural Upazilas (subdistricts) in Chandpur district, Bangladesh, located approximately 55 km southeast of the capital Dhaka. The International Centre for Diarrhoeal Disease Research, Bangladesh (icddr,b) has been operating the Health and Demographic Surveillance System (HDSS) in the area since 1966. The current population size is roughly 230 000.^20^ In the HDSS area, trained Community Health Research Workers (CHRWs) collect important events such as marriage, birth, death and migration (both out and in), and selected morbidity data from women of reproductive age (15–49 years) and children less than 5 years of age through bimonthly household visits.

Data for this paper were collected in the HDSS area with two separate service zones: the icddr,b service area and the government service area. In the icddr,b service area, women and children receive healthcare from providers employed by icddr,b. It is divided into four administrative blocks, with each block covering a population of about 27 000.^21^ Each block provides 24-hour delivery care at a subcentre staffed by midwives. Clinical activities in the subcentre are supported by a hospital in Matlab Township, staffed by medical doctors and nurses who offer basic obstetric care. The icddr,b Matlab Hospital does not have a CS facility; therefore, pregnant women who need CS usually refer to Upazila Health Complex, run by the government, or to a private clinic with a CS facility available. In the government service area, the residents receive care from government healthcare providers. The government area provides services through a three-tiered system made up of community clinics, union health facilities and family welfare centres, which are all linked with the Upazila Health Complex, located in Matlab Municipality. The Upazila Health Complex has been offering CS for more than 15 years; however, due to the lack of anaesthetists or obstetricians, services were often interrupted throughout the years. Since 2010, a number of for-profit private facilities have been established in the study area, and those clinics are mostly engaged in providing CS delivery in the study area.^17 22^ Before this private initiative, women who needed a CS had to travel to the neighbouring districts—Chandpur, Comilla and Narayanganj, which are located in the north and north-east regions of Matlab.

### Study design and participants

This cohort study used the information collected by the ongoing HDSS in Matlab. We evaluated the socioeconomic and demographic factors associated with CS using prospective data from icddr,b and government service areas from 2005 to 2019. The study sample included all women who appeared in the HDSS databases, were identified as pregnant during the household visits of CHRWs and delivered live-born or stillborn babies.

### Data collection

In the study area, CHRWs collected pregnancy and delivery-related information during regular home visits every 2 months. The CHRWs asked the women of reproductive age whether their last menstrual period was overdue for more than 2 weeks and offered a pregnancy test after receiving a positive response. The confirmed pregnancies, afterwards, were observed, and information related to pregnancy outcomes and delivery places was collected at each bimonthly household visit.

#### Dependent variable

The dependent variable is CS delivery, and it was dichotomous (coded as 1 if the respondents underwent CS delivery and 0 if otherwise).

#### Independent variables

Detailed background characteristics about each woman, such as age, parity, education level, asset score and gestational age, were collected. Maternal age was categorised into <20, 20–24, 25–34 and ≥35 years; parity into 0, 1–2, ≥3; and education into no education, primary education and secondary and above. Wealth status was assessed by generating scores through principal components analysis based on ownership of household assets, such as consumer items, house characteristics, type of drinking water and toilet facilities. The generated scores were divided into quintiles, where 1 represented the poorest and 5 the wealthiest.^23^ Gestational age was categorised by preterm (<37 gestation weeks at delivery) and term birth (≥37 gestation weeks of age). The place of residence was recorded as taking place at either the icddr,b service area or the government service area.

### Data analysis

Descriptive analysis was conducted in order to understand the characteristics of the study participants. The associations between the independent variables of interest and the outcome variable, CS delivery, were explored in crude and covariate-adjusted logistic regression models. To measure the extent of divergence or convergence in disparities in CS over time, we test interactions between sociodemographic covariates and time. Initial models identified a strong linear trend relationship between the year of birth and the log odds of having CS, with time measures as years since the base year 2005. This also allowed us to model interaction effects as a simple product of the sociodemographic risk category and years since 2005, producing easily interpretable interaction effects. In the interaction analysis, when the adjusted odds ratio (OR) was found to be <1 in a category compared with the reference group, it was considered a negative interaction (meaning convergence between groups). However, when the adjusted OR was found to be >1, it was considered a positive interaction (meaning divergence between groups). Stata V.16 was used for the analysis, and the findings were presented in tables with summary statistics at 95% CIs.

## Results

In total, out of 89 018 pregnancies identified, 77 321 resulted in live births and stillbirths (online supplemental figure S). After excluding the births with no covariate information (age, education, wealth status or delivery type), in total 73 119 women delivered during the study period were included in the analysis. Furthermore, out of the deliveries included in the analysis, 36 924 women delivered in the icddr,b service area, and 36 195 women delivered in the government service area during 2005–2019 (table 1).

**Table 1:** Sociodemographic characteristics of study participants and their associations with caesarean section in Matlab, Bangladesh, 2005–2019

Variable	Total (n=73 119)		Logistic regression of caesarean section	
	Births (%)	Caesarean section (%)	Unadjusted OR (95% CI)	P value
Maternal age (years)				
<20	14 522 (19.9)	31.2	1	
20–24	23 267 (31.8)	30.6	0.98 (0.93 to 1.02)	0.264
25–29	18 493 (25.3)	28.9	0.90 (0.86 to 0.94)	<0.001
30–34	11 143 (15.2)	27.1	0.82 (0.78 to 0.87)	<0.001
≥35	5694 (7.8)	25.6	0.76 (0.71 to 0.81)	<0.001
Level of education				
No education	9395 (12.8)	15.9	1	
Primary education	15 385 (21.0)	17.0	1.09 (1.02 to 1.17)	0.016
Secondary or above	48 339 (66.1)	35.9	2.97 (2.81 to 3.15)	<0.001
Asset quintiles				
Lowest	13 652 (18.7)	18.6	1	
Lower	12 834 (17.6)	22.7	1.28 (1.21 to 1.36)	<0.001
Middle	14 477 (19.8)	28.4	1.74 (1.64 to 1.84)	<0.001
Richer	16 138 (22.1)	33.4	2.19 (2.1 to 2.31)	<0.001
Richest	16 018 (21.9)	40.8	3.02 (2.87 to 3.19)	<0.001
Parity				
0	28 290 (38.7)	35.4	1	
1–2	36 221 (49.5)	27.9	0.71 (0.69 to 0.73)	<0.001
≥3	8608 (11.8)	15.8	0.34 (0.32 to 0.37)	<0.001
Gestational age (in weeks)				
<37	9832 (13.4)	28.4	1	
≥37	63 287 (86.6)	29.5	1.05 (1.0 to 1.11)	0.029
Place of residence				
Government service area	36 195 (49.5)	27.9	1	
icddr,b service area	36 924 (50.5)	30.8	1.15 (1.12 to 1.19)	<0.001

icddr,b, International Centre for Diarrhoeal Disease Research, Bangladesh.

### Sociodemographic characteristics

Women aged 35 and older contributed the least proportion of the study participants (7.8%), the 20–24 and 25–29 age groups formed the highest proportion (31.8% and 25.3%), followed by the 30–34 group and those younger than 20 year’s groups, which accounted for 15.2% and 19.9% of the total sample, respectively. Women with a level of education secondary or above accounted for the majority of participants (66.1%). Participants with a parity of zero formed 38.7% of participants, while 49.5% had a parity of one or two and 11.8% had a parity of three or more. The majority of participants had a gestational age of 37 weeks or more at delivery (86.6%) (table 1).

Table 1 also presents bivariate analyses of CS frequency in terms of these sociodemographic characteristics over the study period. The probability of CS was lower at older ages, with women aged ≥35 having 24% lower odds of CS than women under age 20 (OR: 0.76, 95% CI 0.71 to 0.81). Women with higher levels of education were more likely to have CS, with those having secondary schooling having nearly three times higher odds of CS than those with no schooling (OR: 2.97, 95% CI 2.81 to 3.15). Assets were also strongly associated with CS, with all asset quintiles significantly different from the lowest and the top quintile being three times more likely than the lowest group (OR: 3.02, 95% CI 2.87 to 3.19). CS was less likely at higher parity, with about 66% likelihood of CS among third births or higher versus first births (OR: 0.34, 95% CI 0.32 to 0.37). CS was more likely for births≥37 weeks’ gestational age (OR: 1.05, 95% CI 1.0 to 1.11). CS was more common in the icddr,b service area than the government service area (OR: 1.15, 95% CI 1.12 to 1.19).

### CS delivery trends over time

**Figure 1:**
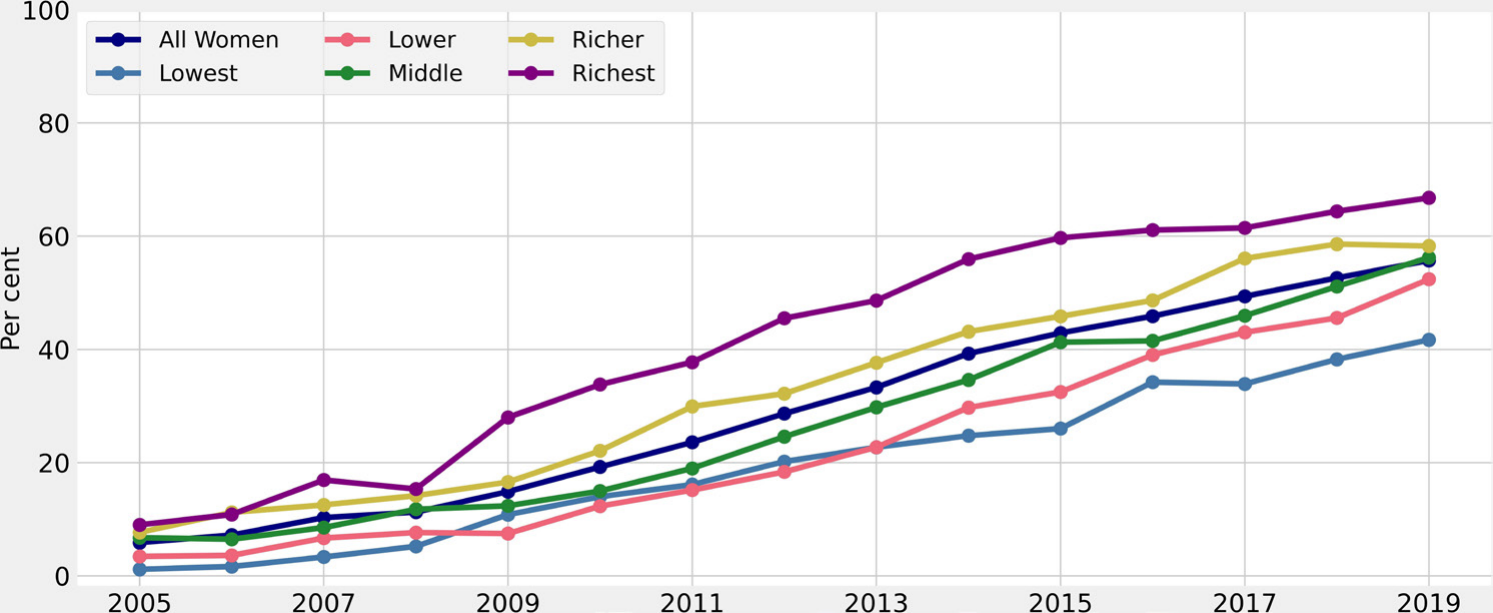
Percentage of births with caesarean delivery over time by asset, 2005–2019 in Matlab, Bangladesh.

The proportion of CS was measured out of the total population, irrespective of whether the women had a single or multiple CS. Out of the total 16 812 women who underwent CS, 12 518 women had a single CS, and 4294 women had multiple CSs (n=8967). The proportions of CS among all study participants by year and by service areas are presented in Table S. The CS procedures have steadily increased across all groups from 2005 to 2019. The proportion of births with CS delivery has increased more than ninefold, from 5.9% in 2005 to 55.7% in 2019, implying a 3.3% annual growth rate (figure 1, online supplemental Table S). For women with the ‘richest’ asset score, CS has increased about sevenfold, from 9% in 2005 to 66.8% in 2019. Starting from the lowest asset level, CS has increased about 41-fold for the lowest asset category, from 1.1% in 2005 to 42% in 2019 (figure 1).

### Determinants of CS

Table 2 presents the associations between sociodemographic factors and CS delivery. There was a significant association between maternal age, level of education and asset score with CS. After adjusting for parity and other covariates, we found that the likelihood of CS increased strongly and significantly at higher ages. Women aged 30–34 years and ≥35 years were about 2.5 times more likely to deliver via CS compared with women aged <20 years. The odds of CS rose with increased levels of education. Women with secondary education or above were one and a half times more likely to have a CS than women with no formal education (OR: 1.48, 95% CI 1.39 to 1.58). Lastly, as a woman’s wealth score increased, so too did her chances of delivering via CS. Women in the ‘richer’ category were about 83% more likely (OR: 1.83, 95% CI 1.73 to 1.95), and women in the ‘richest’ category were about three times as likely (OR: 2.71, 95% CI 2.55 to 2.87) to have a CS than women in the ‘lowest’ wealth quintile. The odds of CS were significantly lower at higher parities, with third or higher-order births 69% less likely to have CS as first births (OR: 0.31, 95% CI 0.29 to 0.33). The likelihood of CS increased linearly with increased year of birth, by about 24% per year (OR: 1.24, 95% CI 1.23 to 1.25). We tested alternate models with year of birth dummies, and the linear term provided a better fit by all information criteria.

**Table 2:** Association of sociodemographic variables and time with caesarean section of pregnancy cohorts 2005–2019 in Matlab, Bangladesh

Variable	Adjusted OR (95% CI)	P value
Maternal age in years (ref≤20)		
20–24	1.39 (1.32 to 1.47)	0.001
25–29	1.83 (1.72 to 1.95)	<0.001
30–34	2.42 (2.24 to 2.61)	<0.001
≥35	2.89 (2.62 to 3.19)	<0.001
Level of education (ref=no education)		
Primary education	0.92 (0.86 to 1.00)	0.027
Secondary or above	1.48 (1.39 to 1.58)	<0.001
Asset score (ref=lowest quintile)		
Lower	1.12 (1.05 to 1.19)	<0.001
Middle	1.38 (1.29 to 1.46)	<0.001
Richer	1.83 (1.73 to 1.95)	<0.001
Richest	2.71 (2.55 to 2.87)	<0.001
Parity (ref=0)		
1–2	0.59 (0.56 to 0.62)	<0.001
≥3	0.31 (0.29 to 0.33)	<0.001
Gestational age (ref<37 weeks)		
≥37 weeks	0.88 (0.83 to 0.93)	<0.001
Place of delivery (ref=government service area)		
icddr,b service area	1.18 (1.14 to 1.22)	<0.001
Observation year		
Birth year	1.24 (1.23 to 1.25)	<0.001

icddr,b, International Centre for Diarrhoeal Disease Research, Bangladesh.

**Figure 2:**
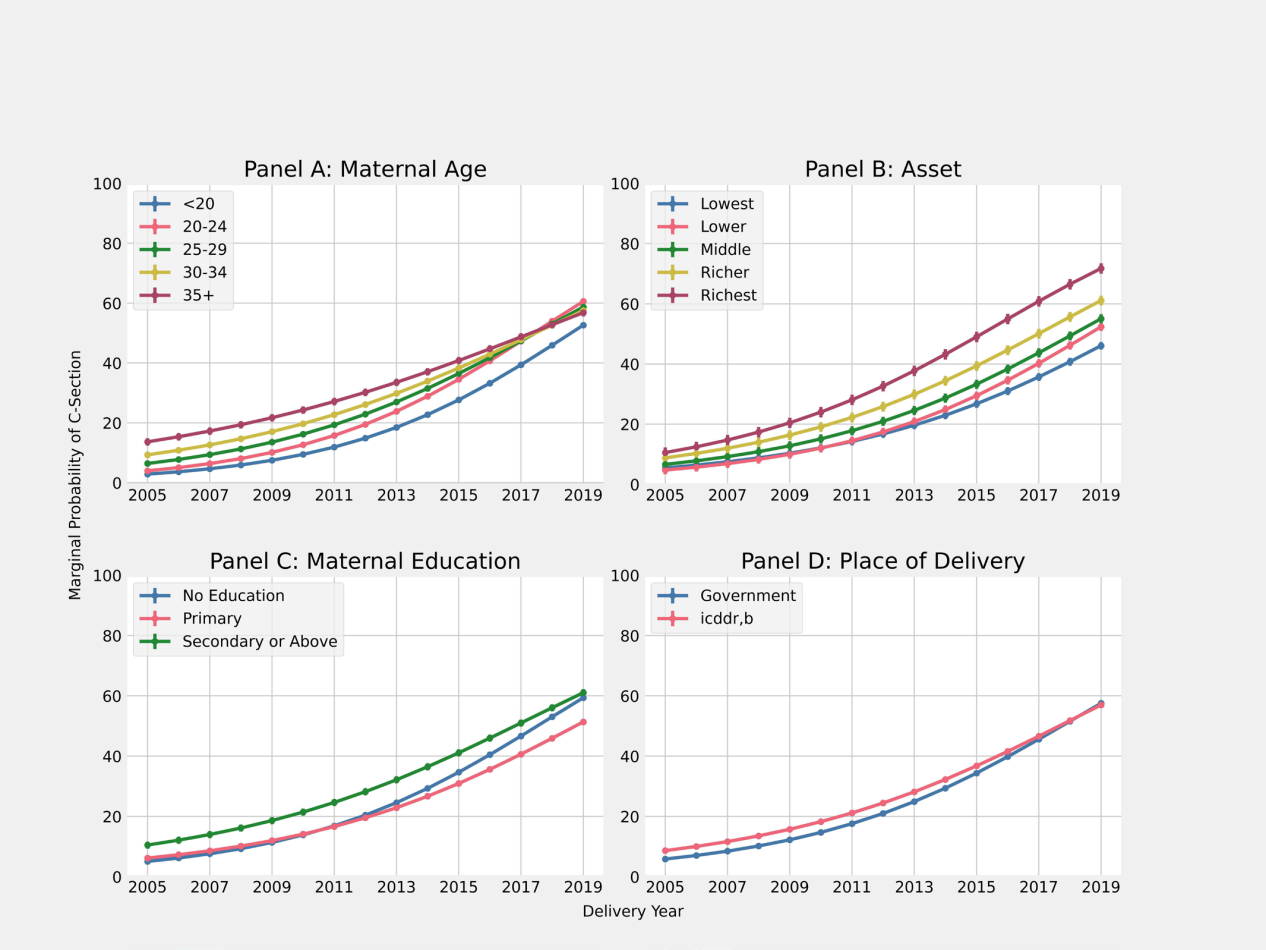
Predictive marginal probability of caesarean section based on model with year of birth interactions. icddr,b, International Centre for Diarrhoeal Disease Research, Bangladesh.

Further, we tested interactions between sociodemographic factors and birth year, as reported in table 3. Several interactions were highly significant, and the resulting predictive marginal probabilities of CS are shown in figure 2. Looking first at mother’s age, we found significant negative interactions between older age categories and year of birth. For example, in the base year, mothers aged ≥35 were about six times more likely to have CS than mothers aged <20 (main effect OR: 5.94, 95% CI 4.75 to 7.44), but the significant interaction term indicates a convergence of the age pattern of CS over time. Figure 2A indicates that by 2017, the covariate-adjusted CS probability for age 30–34 had already equalled that of women aged ≥35, with compression of variation across the age continuum.

**Table 3:** Interactions between sociodemographic factors and birth year showing the temporal trends of caesarean section in Matlab, Bangladesh

Variable	Main effects		Interaction with year	
	OR (95% CI)	P value	OR (95% CI)	P value
Maternal age (ref<20 years)				
20–24	1.42 (1.25 to 1.61)	<0.001	1.0 (0.99 to 1.01)	0.621
25–29	2.4 (2.07 to 2.79)	<0.001	0.96 (0.94 to 0.97)	<0.001
30–34	3.7 (3.09 to 4.42)	<0.001	0.92 (0.91 to 0.94)	<0.001
≥35	5.94 (4.75 to 7.44)	<0.001	0.89 (0.87 to 0.91)	<0.001
Level of education (ref=no education)				
Primary education	1.25 (1.06 to 1.49)	0.009	0.96 (0.94 to 0.98)	<0.001
Secondary or above	2.36 (2.02 to 2.76)	<0.001	0.94 (0.93 to 0.96)	<0.001
Asset score (ref=lowest quintile)				
Lower	0.85 (0.72 to 1.01)	0.072	1.03 (1.01 to 1.05)	0.001
Middle	1.24 (1.06 to 1.46)	0.009	1.01 (0.99 to 1.03)	0.195
Richer	1.74 (1.5 to 2.02)	<0.001	1.01 (0.99 to 1.02)	0.457
Richest	2.17 (1.88 to 2.52)	<0.001	1.03 (1.01 to 1.04)	0.002
Parity (ref=0)				
1–2	0.35 (0.32 to 0.39)	<0.001	1.05 (1.04 to 1.06)	<0.001
≥3	0.16 (0.13 to 0.2)	<0.001	1.09 (1.07 to 1.12)	<0.001
Gestational age (ref<37 weeks)				
≥37 weeks	1.22 (1.07 to 1.38)	0.003	0.96 (0.95 to 0.98)	<0.001
Place of residence (ref=government service area)				
icddr,b service area	1.58 (1.45 to 1.73)	<0.001	0.97 (0.96 to 0.98)	<0.001
Observation year				
Birth year	1.35 (1.32 to 1.39)	<0.001		

icddr,b, International Centre for Diarrhoeal Disease Research, Bangladesh.

Looking at assets, we observed the persistence of strong asset gradients over time. The main effects for all the middle, richer and richest wealth quintiles relative to the lowest quintile were all significant. We found significant positive interactions only for the lower wealth quintile (OR: 1.03, 95% CI 1.01 to 1.05). As a result, figure 2B shows a convergence of CS probabilities among the lower 60% of the asset distribution, but a persistently higher probability of CS in the top asset quintile.

Patterns of temporal variation by education look quite different. The main effect of education was significant for both primary education (OR: 1.25, 95% CI 1.06 to 1.49) and for women with secondary schooling (OR: 2.36, 95% CI 2.02 to 2.76), though much smaller than the asset main effects. Further, we observed a strong negative interaction with year of birth for both primary schooling (OR: 0.96, 95% CI 0.94 to 0.98) and secondary schooling (OR: 0.94, 95% CI 0.93 to 0.96). As a result, the predicted probabilities in figure 2C indicate total convergence of CS probabilities between women with primary and secondary schooling, along with a rapid narrowing of the gap for women with no schooling.

The interaction between place of residence and year of birth is small but significant (OR: 0.97, 95% CI 0.96 to 0.98). The main effect indicates that the odds of CS were 59% higher in the treatment versus the government service area in 2005 (main effect OR 1.58, 95% CI 1.45 to 1.73). But by 2019, the relative risk of CS in treatment versus comparison was close. We also tested the year of birth with gestational age and parity, which were not significant.

## Discussion

Amid rising concern regarding the overuse of CS in less developed countries, our results show that these trends continue nearly unabated across most age, wealth and education groups.

Our analysis of 73 119 births over the course of 15 years revealed that CS delivery in Matlab is both widespread and subject to continuous growth. In the final year of observation in 2019, about 57% of births were delivered by CS, reflecting a nearly ninefold increase over the study period and a 3.3% increase just over the previous year. Among factors positively associated with CS, we observed negative time interactions for maternal age, education and place of delivery, suggesting a convergence in CS rates between high-risk and low-risk groups in these categories. However, positive time interactions for parity and asset score indicate that disparities in CS rates persist and are widening over time.

An unabated increase in CS rate is a global public health concern.^24^ Studies reported increasing trends of CS rates even after the population level had already achieved the highest recommended level by the WHO.^3 24^ A recent systematic review also reported increasing trends of CS rates in South Asian countries, and particularly, much higher performance in CS was achieved by Bangladesh compared with the neighbouring countries.^25^ The CS proportion observed in our study is substantially higher than the national CS rate of 36% over the same period.^12 26^ Yet the rate of relative growth over an identical time period is comparable. The higher rate of CS is also consistent with the higher institutional delivery rate in Matlab than in the rest of the country. A recent global review of national CS patterns found that Bangladesh had the world’s highest share of CS deliveries relative to the number of hospital deliveries.^15^

Higher rates of CS in Matlab may be driven in large part by higher levels of institutional delivery in Matlab. The growth of institutional delivery in the icddr,b service area can be directly attributed to the ongoing MNCH intervention, which increased institutional delivery rates to 72.4% by 2009 in the icddr,b service area versus 22% in the government service area.^22^ Yet our analysis of temporal interactions found that rates of CS in the government service area have converged rapidly to those of the icddr,b service area. By 2019, the comparison area had just 2% fewer CSs, meaning that the comparison area was less than a year behind the treatment area’s growth trend. In all likelihood, the icddr,b area served as an accelerant to the rollout of facility delivery and, by extension, CS in the government service area. Furthermore, the ongoing demand-side financing in the government service area might pull women to institutional delivery irrespective of socioeconomic condition and contribute to an increase in the number of CS deliveries in the government service area.^27 28^

Gaps between sociodemographic status groups have been extensively reported from different geographical areas.^25 29 30^ The size and duration of our dataset allowed us to take sociodemographic factors that are highly predictive of CS—age, assets, education and geographical location, and look over time for signs of convergence and divergence. Temporal interactions were often highly significant and negative, and thus pointed to the closing of demographic and social gaps on the path towards the near universality of CS. This is most notable in the case of age. Globally, a medically necessary CS is more likely to be indicated with advanced age due to the higher potential for medical risk factors and pregnancy-related complications that necessitate a non-vaginal birth, including increased risk of eclampsia, prior CS, declining trial of labour after CS, foetal growth restriction, multiple gestation, placenta previa and placenta accreta.^31^ In the present study, the risk increased noticeably in the older age group after adjustment. At older ages, women are more likely to experience higher morbidities, have a history of previous CS and be more involved in decision-making regarding the mode of delivery.^32^,^35^ Yet our results indicate a rapid convergence across all ages, with women aged 30–34 already having a covariate-adjusted CS risk equivalent to women aged ≥35. To be clear, this convergence does not mean that CS growth is slowing down among older women, but rather younger women are rapidly closing the gap even as rates continue to rise among the over-35 population.

Research has indicated that the growing economic burden of paying for a CS in Bangladesh means it may be difficult for women with low asset scores to access this service.^15^ Our analysis does indicate a persistent wealth gap in CS utilisation, with the top 20% and, to some extent, the second 20% of mothers maintaining a continued advantage over lower quintiles. Within the top quintile in 2014, the unadjusted proportion having CS was 54%, and still rising by more than 10% annually. If high-asset women represent the leading edge of CS growth, where we might also witness an eventual flattening out of growth as rates approach some maximum, our results show no sign of any maximum being reached. While prices clearly place some kind of brake on growth for lower asset groups, we nonetheless find that almost 40% of births in even the lowest quintile were by CS in 2019.

Temporal patterns by education groups may offer some clearer signs of convergence. In many societies that previously saw growth in CS, the slowing of CS growth has often come first among better-educated women,^36^ who may be better able to articulate their needs and advocate for themselves, particularly when it comes to their birthing preferences. We do find significant negative interactions between education and time, indicating a relative slowing down of CS growth among the most educated relative to other groups. Indeed, we observe that the covariate-adjusted risk of CS among women with secondary and primary schooling had converged by 2014. Yet this convergence is not accompanied by any real slowing down of the trend for the most educated, but rather an acceleration of growth for women with lower levels of schooling.

The strengths of the present study relied on the prospectively collected delivery information and related sociodemographic data over 15 years in a rural community in Bangladesh. Our study also has a few limitations, which are worthy of mention. Most notably, it was conducted in one rural study area of unusually high penetration. While we would argue that Matlab can serve as a sentinel for potential future trends in other areas, it is also possible that the area is unique for having high availability of CS services. A second limitation relates to the lack of detailed behavioural measures, including women’s preference, that could capture the predisposition to seek CS by the women included in the study. In addition, the present study did not consider clinical risk factors (maternal and fetal) to evaluate increasing trend of CS in Matlab during the study period. While this study provided information that CS rate is rising rapidly, we could not explain the reason. Further, our measure of CS is based on client self-report rather than clinical records, though this limitation is mitigated by the fact that CS is a high salience event and that it is measured no more than 3 months after delivery. Finally, we have not established whether these additional CS deliveries are excess in the sense that they are resulting in unnecessary complications or consequences for perinatal morbidity or mortality. While studies have established the negative consequences of CS in developing countries where the alternative is safe vaginal delivery, the share of births that would benefit from CS may be much higher in Bangladesh, given concerns over the capacity to conduct safe vaginal delivery.^37^

In spite of these limitations, this study provides the clearest evidence to date of the rapid transition towards nearly universal CS delivery in a rural area of Bangladesh with high availability of services. Whether Matlab is truly representative of the entire country or not, these findings offer one potential future for the rest of Bangladesh and for many other developing countries. CS is a procedure that poses many apparent benefits to expectant parents and to healthcare providers, especially in healthcare systems that are trying to achieve universal safe delivery at a fraction of the costs expended in wealthier countries. New studies are needed to establish the potential health risks associated with these patterns and to understand the potential social and institutional factors influencing this trend.

### Conclusions

The observed transition towards near-universal CS, irrespective of sociodemographic status or geographic location, is likely to be the inevitable future for rural Bangladesh. If such high rates of CS are considered suboptimal for mother-and-child health, then evidence is needed to support alternatives.

## Supplementary material

10.1136/bmjgh-2024-018600 online supplemental file 1

## Data Availability

Data are available upon reasonable request.
