# Supplementary material for: Sociodemographic trends associated with caesarean delivery in rural subdistricts of Bangladesh: a cohort study, 2005–2019
Source: BMJ Glob Health. 2025 Dec 19;10(12):e018600. doi: 10.1136/bmjgh-2024-018600 (PMC12716527; doi:10.1136/bmjgh-2024-018600)
Supplement: online supplemental file 1 [file bmjgh-10-12-s001.docx]

Table S. Frequencies of caesarean section rates in Matlab, Bangladesh, 2005-2019.

| Year | Caesarean section n (%) | | |
| --- | --- | --- | --- |
|  | icddr,b service area (N=11383) | Government service area (N=10102) | All  (N=21485) |
| 2005 | 180 (7.1) | 116 (4.6) | 296 (5.9) |
| 2006 | 205 (8.3) | 146 (6.0) | 351 (7.2) |
| 2007 | 282 (12.0) | 198 (8.6) | 480 (10.3) |
| 2008 | 335 (13.7) | 184 (8.5) | 519 (11.2) |
| 2009 | 439 (17.7) | 270 (11.8) | 709 (14.9) |
| 2010 | 582 (22.7) | 368 (15.5) | 950 (19.2) |
| 2011 | 678 (26.7) | 473 (20.2) | 1151 (23.6) |
| 2012 | 841 (32.2) | 561 (24.6) | 1402 (28.7) |
| 2013 | 906 (35.5) | 686 (30.7) | 1592 (33.3) |
| 2014 | 1095 (41.9) | 864 (36.4) | 1959 (39.3) |
| 2015 | 1006 (42.2) | 1218 (43.5) | 2224 (42.9) |
| 2016 | 1109 (45.6) | 1325 (46.1) | 2434 (45.9) |
| 2017 | 1146 (50.6) | 1308 (48.4) | 2454 (49.4) |
| 2018 | 1392 (52.9) | 1300 (52.3) | 2692 (52.6) |
| 2019 | 1187 (56.8) | 1085 (54.6) | 2272 (55.7) |

All livebirths and stillbirths

2005-2019

(n=77321)

- Age missing (n=1725)
- Education missing (n=96)
- Asset score missing (n=2233)
- Delivery mode missing (n=148)

Total livebirths and stillbirths included in the analysis

(n=73119)

All pregnancies in HDSS

2005-2019

(n=89018)

- Induced abortion (n=3663)
- Spontaneous abortion (n=8034)

Figure S: Study flowchart.
